# Editorial: Understanding the Heterogeneity in Exercise-Induced Changes in Glucose Metabolism to Help Optimize Treatment Outcomes

**DOI:** 10.3389/fendo.2021.699354

**Published:** 2021-05-27

**Authors:** Thomas P. J. Solomon, John P. Thyfault, Jacob M. Haus, Kristian Karstoft

**Affiliations:** ^1^ Blazon Scientific, London, United Kingdom; ^2^ Departments of Molecular & Integrative Physiology and Internal Medicine, University of Kansas Medical Center, Kansas City, KS, United States; ^3^ School of Kinesiology, University of Michigan, Ann Arbor, MI, United States; ^4^ Centre for Physical Activity Research, Rigshospitalet, Copenhagen, Denmark

**Keywords:** exercise training, heterogeneity, variability, inter-individual, glucose control, metformin, hyperglycemia, diabetes

Regular exercise improves several aspects of glucose metabolism. The evidence to date has culminated in clear public health physical activity guidelines (1). However, for patients with diabetes, physical activity guidelines are no different from those issued for the general population. While many randomized controlled trials (RCTs) have demonstrated the benefits of exercise for the prevention and the treatment of diabetes, some trials have not and several studies now highlight the large variability that exists in the inter-individual changes in blood glucose control following exercise (2–5). A 2018 narrative review (6) speculated that this variability is explained by exercise dose, meal-exercise timing, drug-exercise interactions, and more. But these speculations were largely based on observational and correlative evidence because prospective trials were lacking. Identifying and understanding the causes of this *response heterogeneity* is critical for maximizing therapeutic outcomes for patients with, or at risk of, diabetes. Optimizing the therapeutic effect of exercise may also reduce diabetes incidence, complications, and health care burdens. Therefore, this Research Topic aimed to publish papers that: (i) advance our understanding of the inter-individual heterogeneity of exercise-induced changes in blood glucose control, (ii) identify factors influencing such heterogeneity, and (iii) test the causality of such factors.

## What Did This Topic Find?

We accepted ten papers from experts in the field. Three focussed on exercise intensity, exercise duration, and exercise type. For example, the meta-analysis by Liu et al. found that high-intensity interval training better improves blood glucose in children and adolescents with obesity when compared to moderate-intensity continuous training. The narrative review by Paquin et al. argued for the use of resistance exercise protocols aimed at invoking both a high oxygen demand and improves muscle function for inducing the greatest muscle adaptations favouring glucose control. The narrative review by Warner et al. examined the impact of exercise on the liver, highlighting the urgent need for clinical studies to unravel the complexity of hepatic glucose metabolism. They argued that the heterogeneity in exercise effects on hepatic insulin sensitivity and splanchnic glucose metabolism in patients with type 2 diabetes may be attributable to between-study variations in exercise mode, duration, intensity, and weight loss. Further to the review by Warner et al.; Brennan et al. completed an RCT to objectively characterise the inter-individual heterogeneity of several health-related variables in response to energy restriction-induced weight loss with or without exercise, in older-aged adults with obesity. They found that the addition of exercise to energy restriction-induced weight loss increased the proportion of patients showing improvements in blood glucose control and cardiometabolic risk compared to weight loss alone.

To help deepen our understanding of *response heterogeneity*, Munan et al. completed a meta-analysis of single-bout and training studies that used continuous glucose monitoring (CGM) to assess glucose control. The meta-analysis showed that acute exercise and short-term training is sufficient to improve 24-hour glucose profiles in adults with type 2 diabetes but that there is high inter-individual heterogeneity, which was explained in part by the sex of participants, the timing of exercise, and the extent to which glycaemia is impaired on non-exercise days. Two RCTs included in this topic helped probe the causality of these sources of *response heterogeneity*. The RCT by Carter and Solomon showed that experimentally-induced pre-exercise hyperglycaemia blunted the glucoregulatory benefits of a single exercise bout, while the RCT by Porter et al. examined exercise-meal timing, finding that moderate-intensity exercise after an evening meal caused transient asymptomatic hypoglycaemia to a greater extent in women with diabetes than in men.

Finally, three papers in our topic focussed on drug-exercise interactions. The narrative review by Pitt et al. discussed the pharmacokinetics of subcutaneously-administered insulin in the context of type 1 diabetes, contending that exercise may increase circulating insulin concentrations and therefore contribute to exercise-related hyperinsulinemia and consequent hypoglycaemia in insulin-dependent patients. They argued that the location and depth of insulin injection cause variability in insulin absorption rates, which are influenced during exercise by several factors that must be studied in prospective trials. The narrative review by Malin and Stewart postulated that while metformin attenuates the insulin-sensitising effect of exercise, it has variable outcomes on exercise-induced changes in blood glucose control (i.e., HbA1c). Given that metformin is not always used in isolation and given that other medications used to treat diabetes (inc. GLP-1 receptor agonists and SGLT-2 inhibitors) may also interact with exercise, Malin and Stewart emphasised the urgent need for prospective trials in this area. Further to these narrative reviews, Pilmark et al. conducted an RCT to objectively examine the interaction between metformin and exercise. They found that 17 days of metformin treatment increased participants’ ratings of perceived exertion (RPE) during exercise at a fixed intensity but had no effect on self-selected exercise intensity. Therefore, metformin may have implications for exercise adherence but this phenomenon must be prospectively studied in a longer-term trial.

## What Next?

Exercise can be a useful tool for improving glucose control but, for some patients, exercise does not provide the intended therapeutic outcome. It is indeed frustrating for a patient who invests great effort in implementing and maintaining a lifestyle change only to see no obvious benefit to their glucose control. We are a long way from fully understanding *response heterogeneity* to exercise and this topic only scratches the surface in the arduous task of testing the causality of factors responsible. This collection of papers indicates that to help maximise the therapeutic benefit of exercise for all people, we must advance the scientific understanding in this field with basic science mechanistic studies coupled with high-quality long-term RCTs specifically designed to tackle key questions. Namely, the interaction between exercise and glucose-lowering drugs, the interaction between ambient hyperglycaemia and exercise adaptations, and the causal roles of sex, exercise-meal timing, and diurnal timing of exercise on the *response heterogeneity* of blood glucose control deserve increased attention.

## Author Contributions

TS, JT, JH, and KK made substantial contributions to the conception of this topic and the drafting of this editorial. All authors contributed to the article and approved the submitted version.

## Conflict of Interest

TS is the owner of Blazon Scientific company (https://www.blazon-scientific.com).

The remaining authors declare that the research was conducted in the absence of any commercial or financial relationships that could be construed as a potential conflict of interest.
